# Supplementary material for: A population-based study exploring phenotypic clusters and clinical outcomes in stroke using unsupervised machine learning approach
Source: PLOS Digit Health. 2023 Sep 13;2(9):e0000334. doi: 10.1371/journal.pdig.0000334 (PMC10499205; doi:10.1371/journal.pdig.0000334)
Supplement: S1 Text — (DOCX) [file pdig.0000334.s001.docx]

**S1 Text**

## Feature selection

*Least Absolute Shrinkage and Selection Operator (Lasso):* Lasso is a linear regression-based model that is regularized by imposing an L1 penalty on the regression coefficients. The L1 penalty forces the sum of the absolute value of the coefficients to be less than a constant. The variable selection process is embedded in this model because, given the nature of the L1 norm, some coefficients will be forced to be 0, and hence are eliminated from the model.

*Boruta:* Boruta is a random forest-based method that iteratively removes the features that are proven to be statistically less relevant than random probes, which are artificial noise variables introduced in the model by the algorithm.

## Kamila algorithm

The kamila algorithm^1^ is a model-based adaptation of the k-means for managing heterogeneous (mixed) datasets. The Kamila algorithm begins with a set of centroids for the continuous variables and a set of parameters for the categorical variables. For continuous variables, the Euclidean distance with the closest centroid is computed. This set of N minimal distances is used to estimate the mixture distribution of continuous variables. For categorical variables, the probabilities of observing the data given the cluster are computed.

The log-likelihood of the sum of these two components is then used to find the most appropriate cluster for each subject. Based on this temporary partition, the centroids and the parameters are updated to best represent the clusters.

These steps are repeated until the clusters are stable. Finally, multiple runs of this process are performed with different initializations, and the partition maximizing the sum of the best final likelihoods is retained.

## Gradient Boosting Model

The gradient boosting machines algorithm is a boosting algorithm that sequentially combines decision trees such that each additional tree is trained with more weighting placed on correctly predicting data-points that the previous decision trees misclassified.^2^ In simple terms, each new tree aims to correct for the mistakes of the previous trees. Gradient boosting machines aim to minimise the loss function (a measure of difference between the observed and predicted values) by combining a sequence of base-learner models. A common optimisation method to find a minimum is gradient decent which involves going down a gradient to reach a minimum. The key idea behind gradient boosting machines is to sequentially add a new base learner model to the ensemble sequence such that the new model is the model with the greatest correlation with the negative of the loss function’s gradient calculated using the current ensemble sequence predictions.

## SHAP (SHapley Additive exPlanations)

SHAP is a method to explain individual predictions and is based on the game theoretically optimal Shapley values. The goal of SHAP is to explain the prediction of an instance x by computing the contribution of each feature to the prediction. The SHAP explanation method computes Shapley values from coalitional game theory. The feature values of a data instance act as players in a coalition. Shapley values indicate how to fairly distribute the “pay-out” (= the prediction) among the features/variables.

References:

1 Foss AH, Markatou M. kamila: Clustering Mixed-Type Data in R and Hadoop. *J Stat Softw* 2018; **83**: 1–44.

2 Friedman JH. Greedy function approximation: A gradient boosting machine. *Ann Stat* 2001; **29**: 1189–232.
