# Supplementary figures and images for: A population-based study exploring phenotypic clusters and clinical outcomes in stroke using unsupervised machine learning approach
Source: PLOS Digit Health. 2023 Sep 13;2(9):e0000334. doi: 10.1371/journal.pdig.0000334 (PMC10499205; doi:10.1371/journal.pdig.0000334)

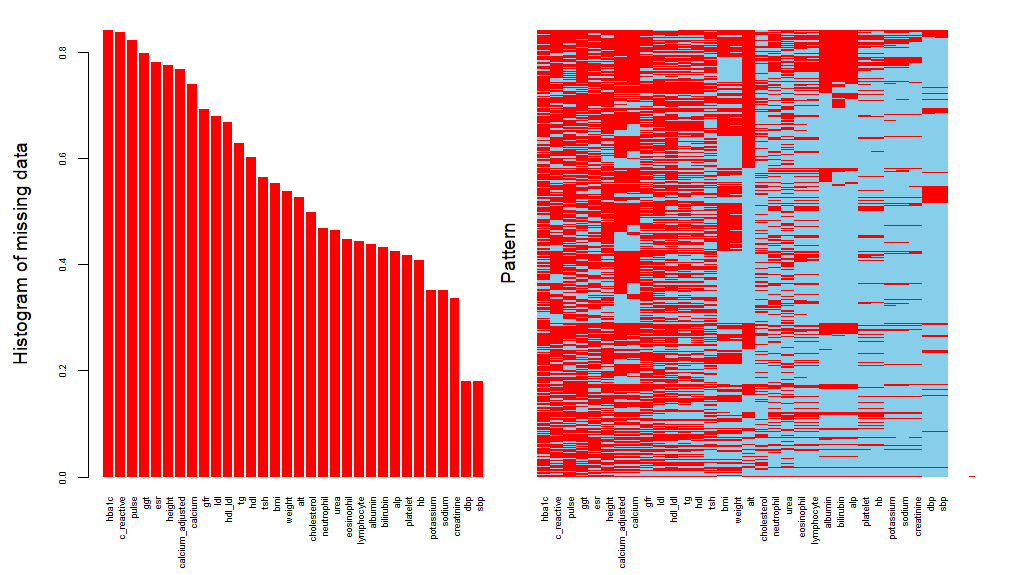
**S1 Fig. All clinical variables with missing values**

Supplement: S1 Fig — (DOCX) [file pdig.0000334.s002.docx]

##
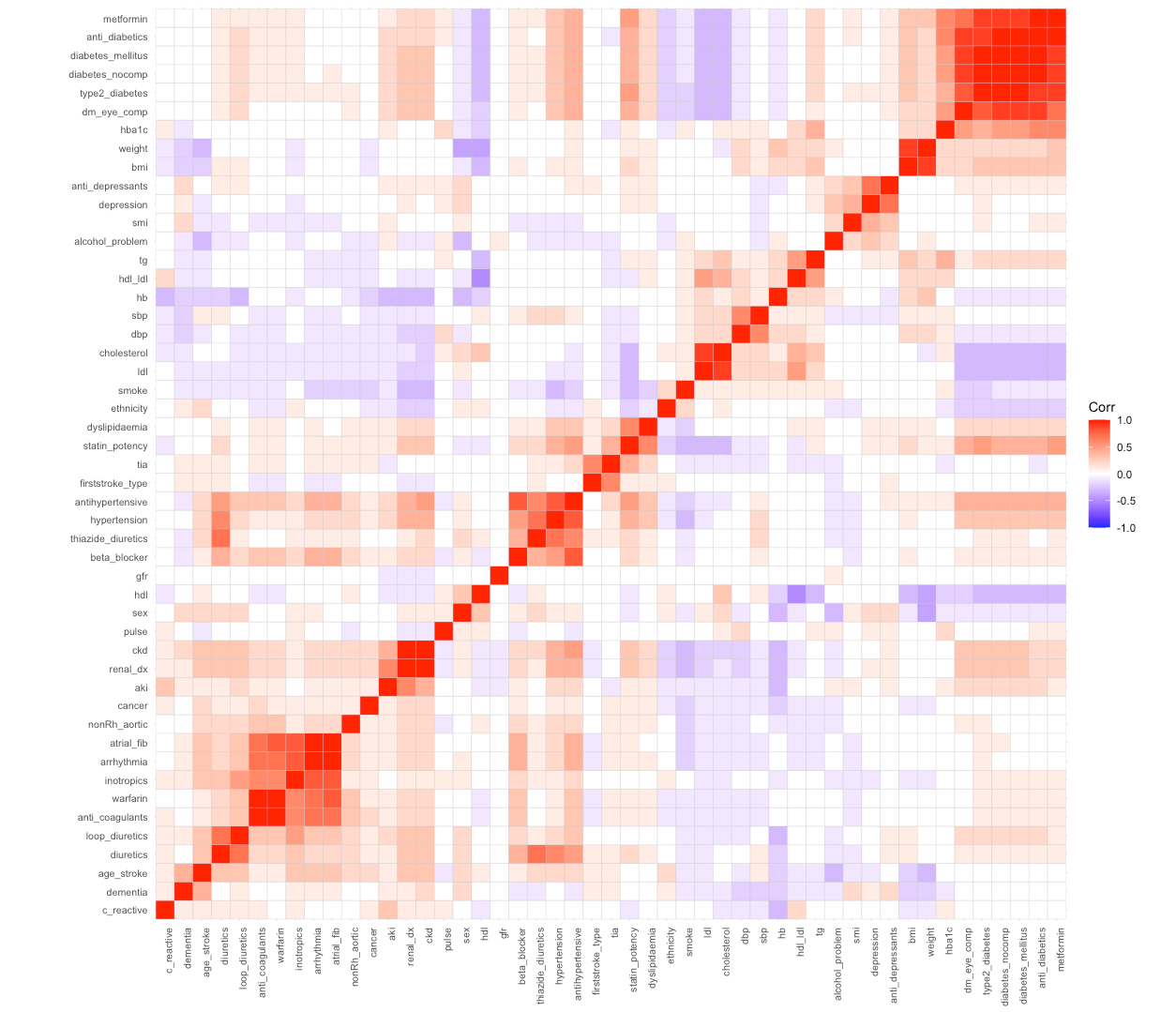
S3 Fig. Plot of correlation matrix of 49 selected variables

Supplement: S3 Fig — (DOCX) [file pdig.0000334.s004.docx]

## S4 Fig. Ranked cross-correlation plot of 49 selected variables


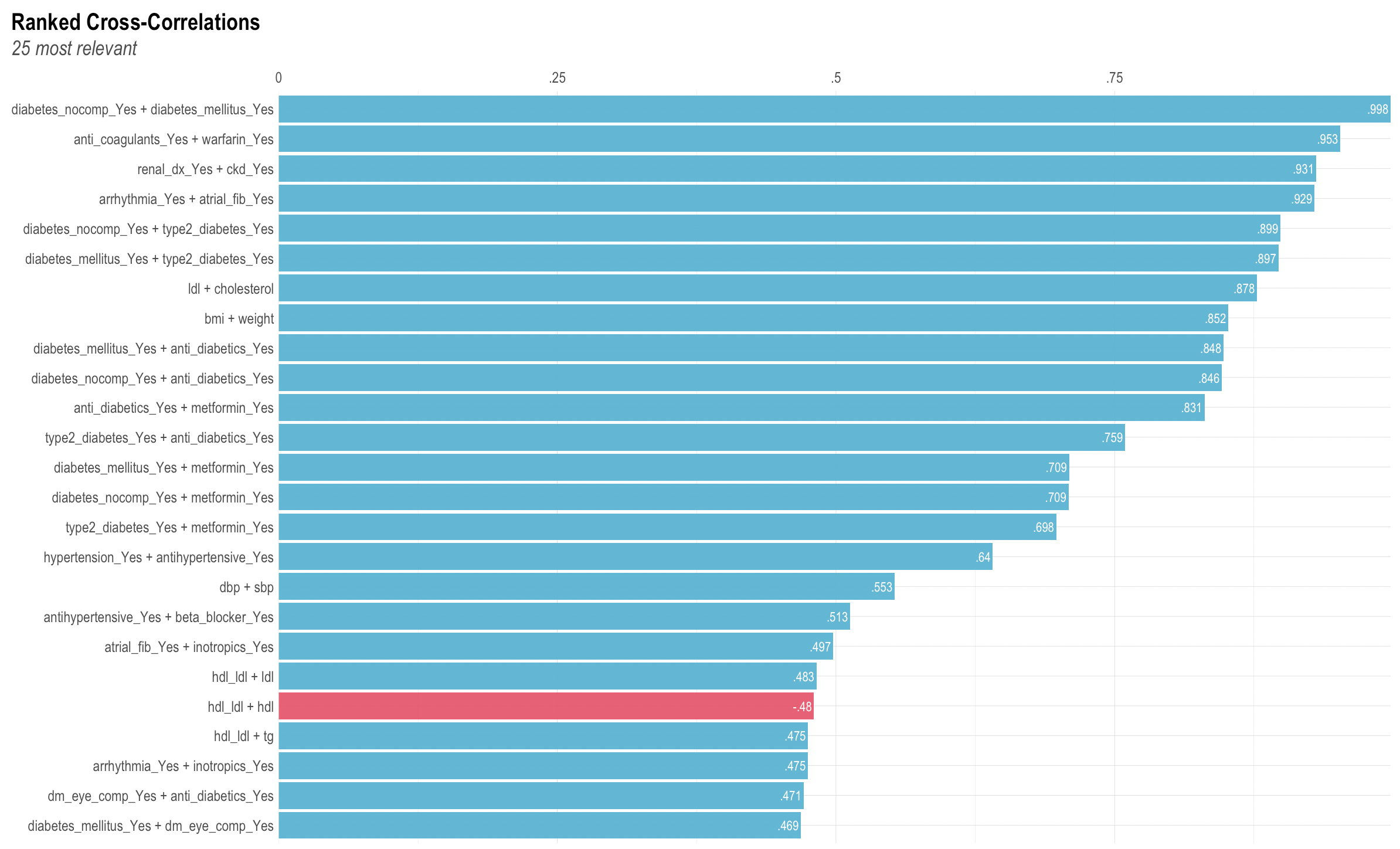

Supplement: S4 Fig — (DOCX) [file pdig.0000334.s005.docx]

## S5 Fig. Optimal number of clusters

1.
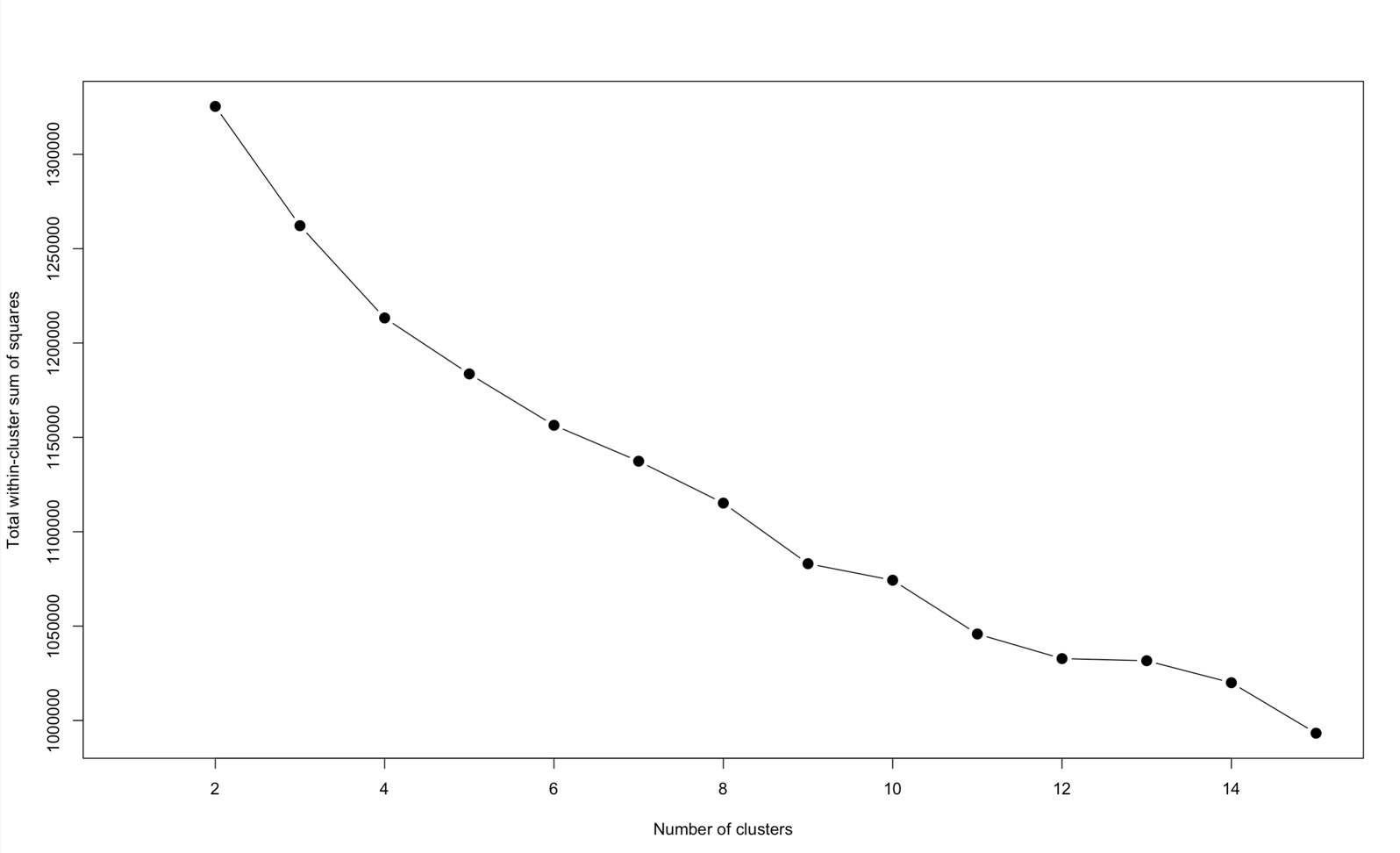
**Elbow method plot**
2.
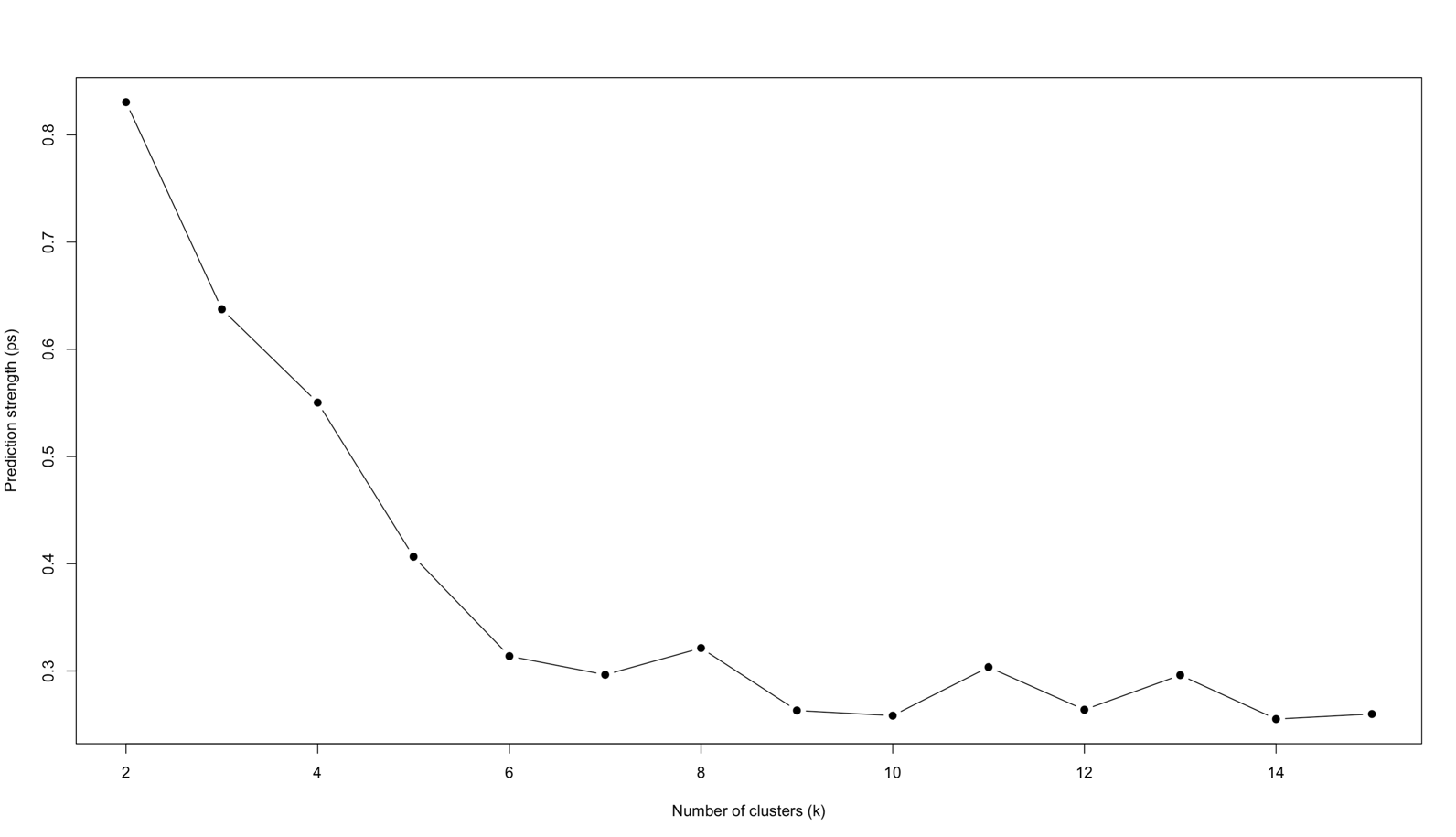
**Prediction strength plot**

Supplement: S5 Fig — (DOCX) [file pdig.0000334.s006.docx]

**S6 Fig. Principal component analysis (PCA) plots**

1. **
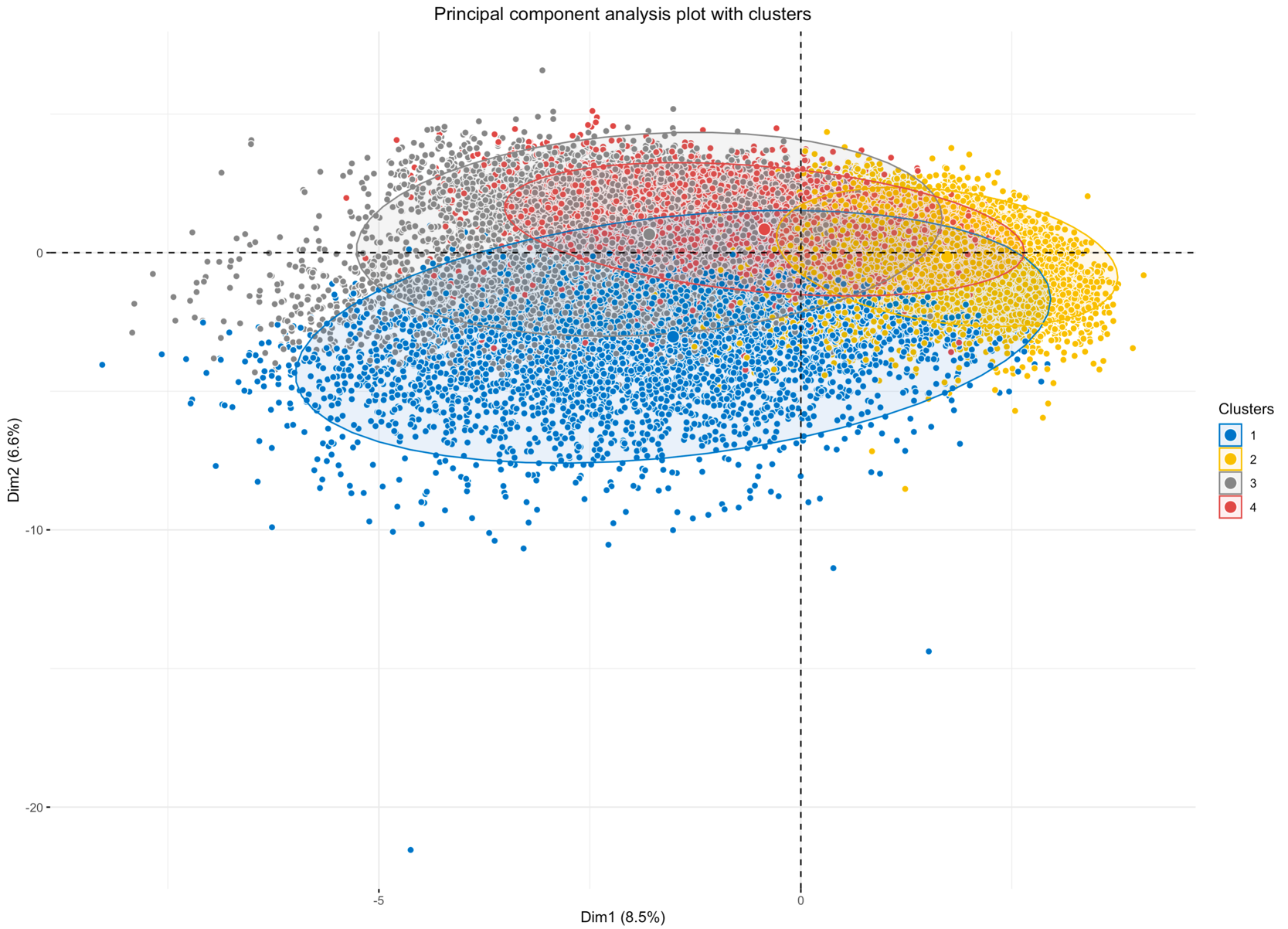
2-dimensional plot showing clusters**

Supplement: S6 Fig — (DOCX) [file pdig.0000334.s007.docx]
