# Supplementary material for: A population-based study exploring phenotypic clusters and clinical outcomes in stroke using unsupervised machine learning approach
Source: PLOS Digit Health. 2023 Sep 13;2(9):e0000334. doi: 10.1371/journal.pdig.0000334 (PMC10499205; doi:10.1371/journal.pdig.0000334)
Supplement: S2 Fig — (DOCX) [file pdig.0000334.s003.docx]

**S2 Fig.** Feature selection

1.
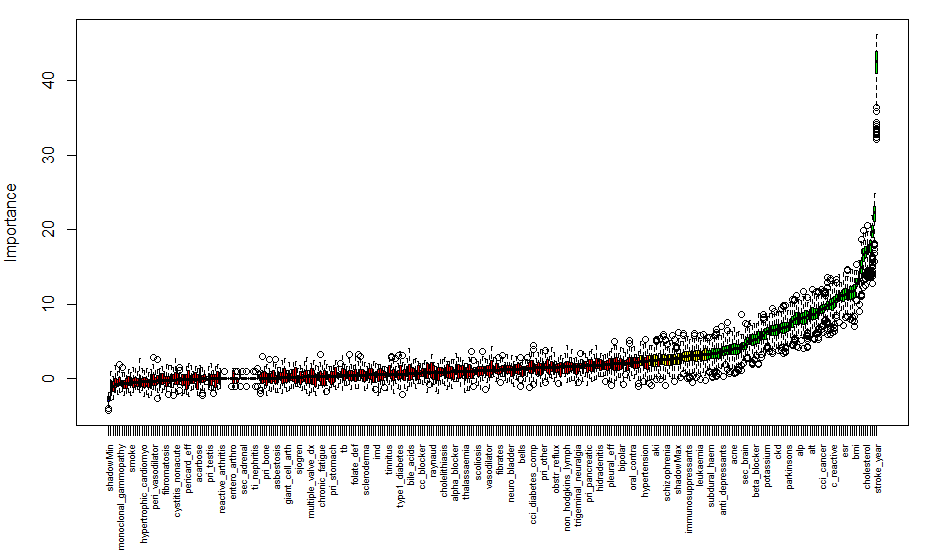
**Boruta – variable importance**

This plot reveals the importance of each of the features. The columns in green are ‘confirmed’ and the one in red are not.

1.
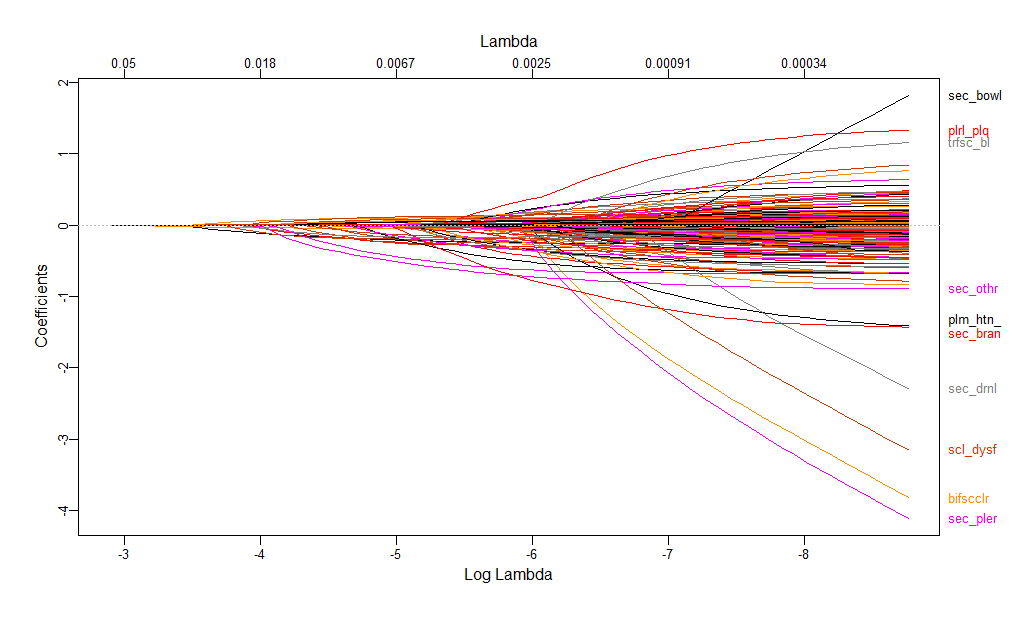
**LASSO regression**

Each curve corresponds to a variable, showing the path of its coefficient against the ℓ1-norm of the whole coefficient vector as λ varies
