# Supplementary material for: A population-based study exploring phenotypic clusters and clinical outcomes in stroke using unsupervised machine learning approach
Source: PLOS Digit Health. 2023 Sep 13;2(9):e0000334. doi: 10.1371/journal.pdig.0000334 (PMC10499205; doi:10.1371/journal.pdig.0000334)
Supplement: S1 Table — (DOCX) [file pdig.0000334.s008.docx]

## S1 Table. Overview of all variables and the in- or exclusion at the various data processing steps

| Variables | Domain | Prevalence, n (%) | LASSO | Boruta | Clinical experts | Selected variables | Cluster analysis |
| --- | --- | --- | --- | --- | --- | --- | --- |
| Sex | Demographics | Men: 31,389 (45.7)  Women: 37,253 (54.3) | X | X | X | X | **X** |
| Age at incident stroke, years | Demographics | Mean: 73.3 (SD: 13.9) | X | X | X | X | **X** |
| Incident stroke sub-type |  | Haemorrhagic: 6,535 (9.5)  Ischaemic: 25,556 (37.2)  Stroke NOS: 36,551 (53.2) | X | X | X | X | **X** |
| Year of incident stroke |  | 1998 – 2017 | X | X |  | X |  |
| Ethnicity | Demographics | Asian: 891 (1.3)  Black: 557 (0.8)  Mixed: 102 (0.1)  Other: 480 (0.7)  White: 60,937 (88.8)  Unknown: 5,675 (8.3) | X | X | X | X | **X** |
| Index of multiple deprivation | Socio-economic status | 1: 14,740 (21.5)  2: 15,289 (22.3)  3: 14,828 (21.6)  4: 12,613 (18.4)  5: 11,056 (16.1)  Unknown: 116 (0.2) |  |  | X |  |  |
| Smoking status | Lifestyle | Never: 26,229 (38.2)  Ex: 16,080 (23.4)  Current: 12,102 (17.6)  Unknown: 14,231 (20.7) | X |  | X | X | **X** |
| Alcohol status | Lifestyle | Yes: 15,822 (23.1)  No: 5,248 (7.6)  Ex: 1,177 (1.7)  Unknown: 46,395 (67.6) |  |  | X |  |  |
| Physical measurements | | | | | | | |
| Body mass index |  | 26.4 (25.1 – 27.9) |  | X | X | X | **X** |
| Diastolic blood pressure | Vital sign | 80 (74 – 84) |  | X | X | X | **X** |
| Systolic blood pressure | Vital sign | 140 (130 – 149) | X | X | X | X | **X** |
| Height |  | 1.7 (1.6 – 1.7) |  |  |  |  |  |
| Pulse | Vital sign | 76 (73 – 79) |  | X | X | X | **X** |
| Weight | Biochemical test | 73.9 (68.0 – 79.5) |  | X | X | X |  |
| Biochemical tests | | | | | | | |
| Alanine aminotransferase | Biochemical test | 22.35 (19.0 – 26.08) |  | X |  |  |  |
| Albumin level | Biochemical test | 40.65 (39.0 – 42.0) |  | X |  |  |  |
| Alkaline phosphatase | Biochemical test | 91.0 (77.8 – 103.0) | X | X |  |  |  |
| Bilirubin level | Biochemical test | 10.55 (9.0 – 12.0) |  | X |  |  |  |
| Calcium level (adjusted) | Biochemical test | 2.34 (2.31 – 2.36) |  | X |  |  |  |
| Calcium level | Biochemical test | 2.34 (2.31 – 2.37) |  | X |  |  |  |
| Creatinine level | Biochemical test | 90.48 (80.0 – 100.0) |  | X |  |  |  |
| C-reactive protein | Biochemical test | 10.0 (6.45 – 15.0) |  | X | X | X | **X** |
| Eosinophil level | Biochemical test | 0.26 (0.16 – 0.38) |  | X |  |  |  |
| Erythrocyte sedimentation rate | Biochemical test | 18.0 (12.9 – 23.03) |  | X |  |  |  |
| Gamma glutamyl transpeptidase | Biochemical test | 43.13 (32.93 – 57.83) |  | X |  |  |  |
| Glomerular filtration rate | Biochemical test | 67.04 (62.23 – 71.90) |  | X | X | X | **X** |
| Haemoglobin level | Biochemical test | 13.53 (12.9 – 14.2) | X | X | X | X | **X** |
| Glycated haemoglobin (hba1c) level | Biochemical test | 50.0 (46.79 – 53.46) | X | X | X | X | **X** |
| HDL/LDL ratio | Biochemical test | 3.65 (3.22 – 4.10) | X | X | X | X |  |
| High-density lipoprotein (HDL) cholesterol | Biochemical test | 1.47 (1.30 – 1.63) |  | X | X | X | **X** |
| Low-density lipoprotein (LDL) cholesterol | Biochemical test | 2.97 (2.66 – 3.26) | X | X | X | X | **X** |
| Lymphocyte count | Biochemical test | 2.40 (1.71 – 3.33) |  | X |  |  |  |
| Neutrophil count | Biochemical test | 4.74 (4.11 – 5.58) |  | X |  |  |  |
| Platelet count | Biochemical test | 248.0 (221.3 – 275.5) |  | X |  |  |  |
| Potassium level | Biochemical test | 4.4 (4.2 – 4.5) |  | X |  |  |  |
| Sodium level | Biochemical test | 139 (138 – 141) |  | X |  |  |  |
| Thyroid stimulating hormone level | Biochemical test | 20.7 (1.79 – 2.32) | X |  |  |  |  |
| Total cholesterol level | Biochemical test | 5.09 (4.70 – 5.45) |  | X | X | X |  |
| Triglyceride level | Biochemical test | 1.43 (1.21 – 1.67) |  | X | X | X | **X** |
| Urea | Biochemical test | 6.3 (5.4 – 7.1) |  | X |  |  |  |
| Comorbid conditions | | | | | | | |
| Benign neoplasm – brain | Benign neoplasm | 303 (0.4) |  | X |  |  |  |
| Benign neoplasm - colon | Benign neoplasm | 1,093 (1.6) |  |  |  |  |  |
| Benign neoplasm - ovary | Benign neoplasm | 493 (0.7) |  |  |  |  |  |
| Benign neoplasm - stomach | Benign neoplasm | 165 (0.2) |  |  |  |  |  |
| Benign neoplasm - uterus | Benign neoplasm | 149 (0.2) |  |  |  |  |  |
| Haemangioma | Benign neoplasm | 520 (0.8) |  |  |  |  |  |
| Leiomyoma | Benign neoplasm | 573 (0.8) |  |  |  |  |  |
| Cancer *(composite)* | Cancers | 11,111 (16.2) | X | X | X | X | **X** |
| Hodgkin Lymphoma | Cancers | 34 (0.0) |  |  |  |  |  |
| Leukaemia | Cancers | 231 (0.3) | X | X |  |  |  |
| Metastatic tumour | Cancers | 333 (0.5) | X | X |  |  |  |
| Monoclonal gammopathy of uncertain significance | Cancers | 142 (0.2) |  |  |  |  |  |
| Myelodysplastic syndrome | Cancers | 139 (0.2) |  |  |  |  |  |
| Non-Hodgkin Lymphoma | Cancers | 294 (0.4) |  |  |  |  |  |
| Non-metastatic cancer | Cancers | 5,955 (8.7) |  |  |  |  |  |
| Plasma cell malignancy | Cancers | 120 (0.2) |  |  |  |  |  |
| Polycythaemia vera | Cancers | 145 (0.2) |  |  |  |  |  |
| Primary malignancy – biliary | Cancers | 20 (0.0) |  |  |  |  |  |
| Primary malignancy – bladder | Cancers | 393 (0.6) |  |  |  |  |  |
| Primary malignancy – bone | Cancers | 16 (0.0) |  |  |  |  |  |
| Primary malignancy – bowel | Cancers | 880 (1.3) |  |  |  |  |  |
| Primary malignancy – brain | Cancers | 114 (0.2) | X | X |  |  |  |
| Primary malignancy – breast | Cancers | 1,599 (2.3) |  |  |  |  |  |
| Primary malignancy – cervical | Cancers | 78 (0.1) |  |  |  |  |  |
| Primary malignancy – kidney | Cancers | 101 (0.1) |  |  |  |  |  |
| Primary malignancy – liver | Cancers | 10 (0.0) |  |  |  |  |  |
| Primary malignancy – lung | Cancers | 342 (0.5) | X | X |  |  |  |
| Primary malignancy – melanoma | Cancers | 544 (0.8) |  |  |  |  |  |
| Primary malignancy – oesophageal | Cancers | 107 (0.2) | X |  |  |  |  |
| Primary malignancy – oropharyngeal | Cancers | 114 (0.2) |  |  |  |  |  |
| Primary malignancy – other | Cancers | 367 (0.5) |  |  |  |  |  |
| Primary malignancy – ovarian | Cancers | 110 (0.2) |  |  |  |  |  |
| Primary malignancy – pancreas | Cancers | 41 (0.1) |  |  |  |  |  |
| Primary malignancy – prostate | Cancers | 1,135 (1.6) |  |  |  |  |  |
| Primary malignancy – skin | Cancers | 4,283 (6.2) |  | X |  |  |  |
| Primary malignancy – stomach | Cancers | 67 (0.1) |  |  |  |  |  |
| Primary malignancy – testis | Cancers | 26 (0.0) |  |  |  |  |  |
| Primary malignancy – thyroid | Cancers | 26 (0.0) |  |  |  |  |  |
| Primary malignancy – uterus | Cancers | 152 (0.2) |  |  |  |  |  |
| Secondary malignancy – bone | Cancers | 59 (0.1) |  |  |  |  |  |
| Secondary malignancy – brain | Cancers | 36 (0.0) | X | X |  |  |  |
| Secondary malignancy – liver | Cancers | 66 (0.1) |  | X |  |  |  |
| Secondary malignancy – lung | Cancers | 27 (0.0) |  |  |  |  |  |
| Secondary malignancy – lymph nodes | Cancers | 30 (0.0) |  |  |  |  |  |
| Secondary malignancy – others | Cancers | 281 (0.4) | X | X |  |  |  |
| Abdominal aortic aneurysm | Diseases – circulatory system | 457 (0.7) |  |  | X |  |  |
| Arrythmia | Diseases – circulatory system | 6,983 (10.2) | X | X | X | X | **X** |
| Atrial fibrillation | Diseases – circulatory system | 6,453 (9.4) | X | X | X | X |  |
| Atrioventricular block, first degree | Diseases – circulatory system | 39 (0.1) |  |  |  |  |  |
| Atrioventricular block, second degree | Diseases – circulatory system | 14 (0.0) |  |  |  |  |  |
| Atrioventricular block, third degree | Diseases – circulatory system | 35 (0.0) |  |  |  |  |  |
| Cardiomyopathy - other | Diseases – circulatory system | 68 (0.1) |  |  | X |  |  |
| Dilated cardiomyopathy | Diseases – circulatory system | 26 (0.0) |  |  | X |  |  |
| Family history of cardiovascular disease | Diseases – circulatory system | 12,299 (17.9) |  |  | X |  |  |
| Family history of coronary heart disease | Diseases – circulatory system | 8,575 (12.5) |  |  | X |  |  |
| Hypertension | Diseases – circulatory system | 31,844 (46.4) | X |  | X | X | **X** |
| Hypertrophic cardiomyopathy | Diseases – circulatory system | 38 (0.1) |  |  | X |  |  |
| Left bundle branch block | Diseases – circulatory system | 88 (0.1) |  |  | X |  |  |
| Multiple valve disorder | Diseases – circulatory system | 104 (0.1) |  |  | X |  |  |
| Non-rheumatic aortic valve disorder | Diseases – circulatory system | 834 (1.2) | X |  | X | X | **X** |
| Non-rheumatic mitral valve disorder | Diseases – circulatory system | 618 (0.9) |  |  | X |  |  |
| Pericardial effusion | Diseases – circulatory system | 35 (0.0) |  |  |  |  |  |
| Primary pulmonary hypertension | Diseases – circulatory system | 52 (0.1) |  |  | X |  |  |
| Raynaud’s disease | Diseases – circulatory system | 752 (1.1) |  |  |  |  |  |
| Rheumatic valve disorder | Diseases – circulatory system | 141 (0.2) |  |  | X |  |  |
| Right bundle branch block | Diseases – circulatory system | 130 (0.2) |  |  | X |  |  |
| Sick sinus syndrome | Diseases – circulatory system | 74 (0.1) |  |  |  |  |  |
| Subarachnoid haemorrhage | Diseases – circulatory system | 477 (0.7) | X | X |  |  |  |
| Subdural haematoma | Diseases – circulatory system | 114 (0.2) |  | X |  |  |  |
| Supraventricular tachycardia | Diseases – circulatory system | 629 (0.9) |  |  | X |  |  |
| Transient ischaemic attack | Diseases – circulatory system | 14,068 (20.5) |  | X | X | X | **X** |
| Venous thrombolism (excluding PR) | Diseases – circulatory system | 1,789 (2.6) |  |  |  |  |  |
| Ventricular tachycardia | Diseases – circulatory system | 64 (0.1) |  |  | X |  |  |
| Alcoholic liver disease | Diseases – digestive system | 260 (0.4) |  |  | X |  |  |
| Autoimmune liver disease | Diseases – digestive system | 55 (0.1) |  |  |  |  |  |
| Barrett’s Oesophagus | Diseases – digestive system | 505 (0.7) |  |  |  |  |  |
| Cholangitis | Diseases – digestive system | 101 (0.1) |  |  |  |  |  |
| Cholecystitis | Diseases – digestive system | 745 (1.1) |  |  |  |  |  |
| Cholelithiasis | Diseases – digestive system | 2,183 (3.2) |  |  |  |  |  |
| Cirrhosis | Diseases – digestive system | 334 (0.5) |  |  |  |  |  |
| Coeliac disease | Diseases – digestive system | 221 (0.3) |  |  |  |  |  |
| Crohn’s disease | Diseases – digestive system | 209 (0.3) |  |  |  |  |  |
| Diverticular disease | Diseases – digestive system | 4,851 (7.1) |  |  |  |  |  |
| Fatty liver | Diseases – digestive system | 56 (0.1) |  |  | X |  |  |
| Gastritis and duodenitis | Diseases – digestive system | 3,680 (5.4) |  | X |  |  |  |
| Gastroesophageal reflux disease | Diseases – digestive system | 6,339 (9.2) |  |  |  |  |  |
| Irritable bowel syndrome | Diseases – digestive system | 3,264 (4.8) |  |  |  |  |  |
| Liver failure | Diseases – digestive system | 43 (0.1) |  |  |  |  |  |
| Mild liver disease | Diseases – digestive system | 212 (0.3) |  |  |  |  |  |
| Moderate-severe liver disease | Diseases – digestive system | 316 (0.5) |  | X |  |  |  |
| Pancreatitis | Diseases – digestive system | 449 (0.6) |  |  |  |  |  |
| Peptic ulcer disease | Diseases – digestive system | 2,633 (3.8) |  |  |  |  |  |
| Peritonitis | Diseases – digestive system | 308 (0.4) |  |  |  |  |  |
| Portal hypertension | Diseases – digestive system | 32 (0.0) |  |  |  |  |  |
| Ulcerative colitis | Diseases – digestive system | 423 (0.6) |  |  |  |  |  |
| Hearing loss | Diseases – Ear | 10,587 (15.4) |  |  |  |  |  |
| Meniere’s disease | Diseases – Ear | 524 (0.8) |  |  |  |  |  |
| Otitis media | Diseases – Ear | 3,616 (5.3) |  |  |  |  |  |
| Tinnitus | Diseases – Ear | 3,023 (4.4) |  |  |  |  |  |
| Cystic fibrosis | Diseases – Endocrine system | 23 (0.0) |  |  |  |  |  |
| Diabetes mellitus | Diseases – Endocrine system | 7,978 (11.6) |  | X | X | X | **X** |
| Diabetes mellitus, Type 1 | Diseases – Endocrine system | 577 (0.8) |  |  | X |  |  |
| Diabetes mellitus, Type 2 | Diseases – Endocrine system | 6,578 (9.6) |  | X | X | X |  |
| Diabetes mellitus, with complications | Diseases – Endocrine system | 1,404 (2.0) |  |  | X |  |  |
| Diabetes mellitus, with no complications | Diseases – Endocrine system | 7,946 (11.6) |  | X | X | X |  |
| Dyslipidaemia | Diseases – Endocrine system | 6,560 (9.6) |  | X | X | X | **X** |
| Family history of hyperlipidaemia | Diseases – Endocrine system | 86 (0.1) |  |  | X |  |  |
| Hyperparathyroidism | Diseases – Endocrine system | 224 (0.3) |  |  |  |  |  |
| Hypoglycaemia-causing disorders | Diseases – Endocrine system | 234 (0.3) |  |  |  |  |  |
| Hypothyroidism | Diseases – Endocrine system | 4,869 (7.1) |  |  |  |  |  |
| Obesity | Diseases – Endocrine system | 3,096 (4.5) |  |  | X |  |  |
| Polycystic ovarian syndrome | Diseases – Endocrine system | 24 (0.0) |  |  | X |  |  |
| Thyroid disease *(hypo or hyperthyroidism)* | Diseases – Endocrine system | 5,708 (8.3) |  |  |  |  |  |
| Anterior uveitis | Diseases – Eye | 694 (1.0) |  |  |  |  |  |
| Blindness | Diseases – Eye | 2,696 (3.9) |  |  |  |  |  |
| Cataract | Diseases – Eye | 10,776 (15.7) | X | X |  |  |  |
| Diabetic ophthalmic complications | Diseases – Eye | 1,986 (2.9) | X |  | X | X | **X** |
| Glaucoma | Diseases – Eye | 3,293 (4.8) |  |  |  |  |  |
| Keratitis | Diseases – Eye | 388 (0.6) |  |  |  |  |  |
| Macular degeneration | Diseases – Eye | 2,307 (3.4) |  |  |  |  |  |
| Posterior uveitis | Diseases – Eye | 47 (0.1) |  |  |  |  |  |
| Retinal detachment | Diseases – Eye | 522 (0.8) |  |  |  |  |  |
| Retinal vascular occlusion | Diseases – Eye | 959 (1.4) |  |  |  |  |  |
| Scleritis | Diseases – Eye | 354 (0.5) |  |  |  |  |  |
| Acute kidney injury | Diseases – genitourinary system | 314 (0.5) |  | X | X | X | **X** |
| Benign prostatic hyperplasia | Diseases – genitourinary system | 4,270 (6.2) |  |  |  |  |  |
| Chronic kidney disease | Diseases – genitourinary system | 7,232 (10.5) |  | X | X | X |  |
| End stage renal disease | Diseases – genitourinary system | 239 (0.4) |  |  | X |  |  |
| Erectile dysfunction | Diseases – genitourinary system | 3,735 (5.4) |  |  | X |  |  |
| Female infertility | Diseases – genitourinary system | 197 (0.3) |  |  |  |  |  |
| Glomerulonephritis | Diseases – genitourinary system | 144 (0.2) |  |  | X |  |  |
| Male infertility | Diseases – genitourinary system | 187 (0.3) |  |  |  |  |  |
| Neuropathic bladder | Diseases – genitourinary system | 1,012 (1.5) |  |  |  |  |  |
| Obstructive and reflux uropathy | Diseases – genitourinary system | 289 (0.4) |  |  |  |  |  |
| Proteinuria | Diseases – genitourinary system | 866 (1.3) |  |  | X |  |  |
| Renal disease | Diseases – genitourinary system | 8,108 (11.8) |  | X | X | X | **X** |
| Urinary incontinence | Diseases – genitourinary system | 4,913 (7.2) | X | X |  |  |  |
| Urolithiasis | Diseases – genitourinary system | 1,599 (2.3) |  |  |  |  |  |
| Allergic and chronic rhinitis | Diseases – respiratory system | 7,024 (10.2) |  |  |  |  |  |
| Asbestosis | Diseases – respiratory system | 117 (0.2) |  |  |  |  |  |
| Asthma | Diseases – respiratory system | 6,771 (9.9) | X |  |  |  |  |
| Bronchiectasis | Diseases – respiratory system | 425 (0.6) |  |  |  |  |  |
| Chronic obstructive pulmonary disease | Diseases – respiratory system | 3,932 (5.7) |  | X |  |  |  |
| Chronic sinusitis | Diseases – respiratory system | 4,357 (6.3) |  |  |  |  |  |
| Pleural effusion | Diseases – respiratory system | 305 (0.4) |  |  |  |  |  |
| Pleural plaque | Diseases – respiratory system | 86 (0.1) |  |  |  |  |  |
| Pneumothorax | Diseases – respiratory system | 228 (0.3) |  |  |  |  |  |
| Pulmonary collapse | Diseases – respiratory system | 69 (0.1) |  |  |  |  |  |
| Pulmonary fibrosis | Diseases – respiratory system | 166 (0.2) | X |  |  |  |  |
| Respiratory failure | Diseases – respiratory system | 28 (0.0) |  |  |  |  |  |
| Sleep apnoea | Diseases – respiratory system | 335 (0.5) |  |  | X |  |  |
| Agranulocytosis | Haem. / Immunological conditions | 272 (0.4) |  |  |  |  |  |
| Anaemia – other | Haem. / Immunological conditions | 4,567 (6.6) |  | X |  |  |  |
| Aplastic anaemia | Haem. / Immunological conditions | 53 (0.1) | X |  |  |  |  |
| Folate deficiency anaemia | Haem. / Immunological conditions | 378 (0.5) |  |  |  |  |  |
| Hypersplenism | Haem. / Immunological conditions | 45 (0.1) |  |  |  |  |  |
| Hyposplenism | Haem. / Immunological conditions | 151 (0.2) |  |  |  |  |  |
| Immunodeficiency | Haem. / Immunological conditions | 17 (0.0) |  |  |  |  |  |
| Iron deficiency anaemia | Haem. / Immunological conditions | 3,023 (4.4) |  |  |  |  |  |
| Other haemolytic anaemia | Haem. / Immunological conditions | 81 (0.1) |  |  |  |  |  |
| Primary thrombocytopaenia | Haem. / Immunological conditions | 83 (0.1) |  |  |  |  |  |
| Sarcoidosis | Haem. / Immunological conditions | 139 (0.2) |  |  |  |  |  |
| Secondary polycythaemia | Haem. / Immunological conditions | 83 (0.1) |  |  |  |  |  |
| Secondary thrombocytopaenia | Haem. / Immunological conditions | 265 (0.4) |  |  |  |  |  |
| Sickle cell trait | Haem. / Immunological conditions | 26 (0.0) |  |  |  |  |  |
| Thalassaemia | Haem. / Immunological conditions | 30 (0.0) |  |  |  |  |  |
| Thalassaemia trait | Haem. / Immunological conditions | 42 (0.1) |  |  |  |  |  |
| Thrombophilia | Haem. / Immunological conditions | 141 (0.2) |  |  | X |  |  |
| Vitamin B12 deficiency anaemia | Haem. / Immunological conditions | 1,682 (2.4) |  |  |  |  |  |
| Chronic viral hepatitis | Infectious diseases | 128 (0.2) |  |  |  |  |  |
| HIV | Infectious diseases | 24 (0.0) |  |  |  |  |  |
| Rheumatic fever | Infectious diseases | 245 (0.4) |  |  |  |  |  |
| Tuberculosis | Infectious diseases | 575 (0.8) |  |  |  |  |  |
| Alcohol misuse | Mental health disorders | 1,903 (2.8) | X | X | X | X | **X** |
| Anxiety | Mental health disorders | 8,782 (12.8) |  |  |  |  |  |
| Autism | Mental health disorders | 18 (0.0) |  |  |  |  |  |
| Bipolar affective disorder | Mental health disorders | 352 (0.5) |  |  | X |  |  |
| Conduct disorder | Mental health disorders | 66 (0.1) |  |  |  |  |  |
| Delirium | Mental health disorders | 567 (0.8) |  |  |  |  |  |
| Dementia | Mental health disorders | 3,532 (5.1) | X | X | X | X | **X** |
| Depression | Mental health disorders | 12,597 (18.4) |  | X | X | X | **X** |
| Eating disorders | Mental health disorders | 62 (0.1) |  |  |  |  |  |
| Hyperkinetic disorders | Mental health disorders | 28 (0.0) |  |  |  |  |  |
| Intellectual disability | Mental health disorders | 264 (0.4) |  |  |  |  |  |
| Insomnia | Mental health disorders | 6,902 (10.1) |  |  |  |  |  |
| Obsessive compulsive disorder | Mental health disorders | 155 (0.2) |  |  |  |  |  |
| Personality disorder | Mental health disorders | 371 (0.5) |  |  |  |  |  |
| Schizophrenia | Mental health disorders | 626 (0.9) |  | X |  |  |  |
| Self-harm | Mental health disorders | 1,495 (2.2) |  | X |  |  |  |
| Severe mental illness | Mental health disorders | 955 (1.4) | X | X | X | X | **X** |
| Substance misuse | Mental health disorders | 701 (1.0) |  |  | X |  |  |
| Ankylosing spondylitis | Musculoskeletal conditions | 102 (0.1) |  |  |  |  |  |
| Back pain | Musculoskeletal conditions | 24,933 (36.3) |  |  |  |  |  |
| Carpal tunnel syndrome | Musculoskeletal conditions | 3,156 (4.6) |  |  |  |  |  |
| Collapsed vertebra | Musculoskeletal conditions | 383 (0.6) |  |  |  |  |  |
| Connective tissue disease | Musculoskeletal conditions | 3,245 (4.7) |  |  |  |  |  |
| Enthesopathy and synovial disorder | Musculoskeletal conditions | 14,198 (20.7) |  |  |  |  |  |
| Fibromatosis | Musculoskeletal conditions | 1,367 (2.0) |  |  |  |  |  |
| Giant cell arteritis | Musculoskeletal conditions | 414 (0.6) |  |  |  |  |  |
| Gout | Musculoskeletal conditions | 3,837 (5.6) |  |  | X |  |  |
| Intervertebral disc disorder | Musculoskeletal conditions | 1,699 (2.5) |  |  |  |  |  |
| Lupus erythematosus | Musculoskeletal conditions | 152 (0.2) |  |  | X |  |  |
| Osteoarthritis | Musculoskeletal conditions | 16,995 (24.8) | X |  |  |  |  |
| Osteoporosis | Musculoskeletal conditions | 4,434 (6.5) |  | X |  |  |  |
| Polymyalgia rheumatica | Musculoskeletal conditions | 1,842 (2.7) |  |  |  |  |  |
| Psoriatic arthritis | Musculoskeletal conditions | 165 (0.2) |  |  |  |  |  |
| Reactive arthritis | Musculoskeletal conditions | 34 (0.0) |  |  |  |  |  |
| Rheumatoid arthritis | Musculoskeletal conditions | 1,317 (1.9) |  |  | X |  |  |
| Scleroderma | Musculoskeletal conditions | 32 (0.0) |  |  |  |  |  |
| Scoliosis | Musculoskeletal conditions | 448 (0.6) |  |  |  |  |  |
| Sjogren syndrome | Musculoskeletal conditions | 123 (0.2) |  |  |  |  |  |
| Spinal stenosis | Musculoskeletal conditions | 597 (0.9) |  |  |  |  |  |
| Spondylolisthesis | Musculoskeletal conditions | 255 (0.4) |  |  |  |  |  |
| Spondylosis | Musculoskeletal conditions | 6,171 (9.0) |  |  |  |  |  |
| Autonomic neuropathy | Neurological conditions | 210 (0.3) |  |  |  |  |  |
| Bell’s palsy | Neurological conditions | 695 (1.0) |  |  |  |  |  |
| Cerebral palsy | Neurological conditions | 64 (0.1) |  |  |  |  |  |
| Chronic fatigue syndrome | Neurological conditions | 1,026 (1.5) |  |  |  |  |  |
| Diabetic neuropathy | Neurological conditions | 409 (0.6) |  |  | X |  |  |
| Epilepsy | Neurological conditions | 1,876 (2.7) | X | X |  |  |  |
| Essential tremor | Neurological conditions | 331 (0.5) |  |  |  |  |  |
| Hemiplegia | Neurological conditions | 342 (0.5) | X |  |  |  |  |
| Migraine | Neurological conditions | 3,610 (5.3) |  |  |  |  |  |
| Motor neurone disease | Neurological conditions | 28 (0.0) |  |  |  |  |  |
| Multiple sclerosis | Neurological conditions | 214 (0.3) |  |  |  |  |  |
| Myasthenia gravis | Neurological conditions | 52 (0.1) |  |  |  |  |  |
| Parkinson’s disease | Neurological conditions | 959 (1.4) | X | X |  |  |  |
| Peripheral neuropathy | Neurological conditions | 1,833 (2.7) |  |  |  |  |  |
| Trigeminal neuralgia | Neurological conditions | 661 (1.0) |  |  |  |  |  |
| Congenital septal defect | Perinatal conditions | 110 (0.2) |  |  |  |  |  |
| Acne | Skin conditions | 693 (1.0) | X | X |  |  |  |
| Actinic keratosis | Skin conditions | 3,400 (5.0) |  |  |  |  |  |
| Alopecia areata | Skin conditions | 148 (0.2) |  |  |  |  |  |
| Dermatitis | Skin conditions | 13,950 (20.3) |  |  |  |  |  |
| Hidradenitis supprativa | Skin conditions | 87 (0.1) |  |  |  |  |  |
| Lichen planus | Skin conditions | 536 (0.8) |  |  |  |  |  |
| Pilonidal cyst/sinus | Skin conditions | 223 (0.3) |  |  |  |  |  |
| Psoriasis | Skin conditions | 2,588 (3.8) |  |  |  |  |  |
| Rosacea | Skin conditions | 1,661 (2.4) |  |  |  |  |  |
| Seborrheic dermatitis | Skin conditions | 3,268 (4.8) |  |  |  |  |  |
| Urticaria | Skin conditions | 2,388 (3.5) |  |  |  |  |  |
| Vitiligo | Skin conditions | 130 (0.2) |  |  |  |  |  |
| Prescribed medications | | | | | | | |
| Acarbose | Prescribed medication | 118 (0.2) |  |  |  |  |  |
| Angiotensin-converting enzyme inhibitor | Prescribed medication | 20,145 (29.3) |  |  | X |  |  |
| Alpha blocker | Prescribed medication | 4,267 (6.2) |  |  |  |  |  |
| Antihypertensive | Prescribed medication | 33,347 (48.6) |  | X | X | X | **X** |
| Antiarrhythmic | Prescribed medication | 3,152 (4.6) |  |  | X |  |  |
| Anticoagulant | Prescribed medication | 4,050 (5.9) |  | X | X | X | **X** |
| Antidepressant | Prescribed medication | 6,368 (9.3) | X | X | X | X | **X** |
| Antidiabetic | Prescribed medication | 15,474 (22.5) |  | X | X | X | **X** |
| Antiepileptic | Prescribed medication | 5,679 (8.3) | X | X |  |  |  |
| Antiplatelet | Prescribed medication | 25,676 (37.4) |  |  | X |  |  |
| Anxiolytic | Prescribed medication | 7,709 (11.2) | X |  |  |  |  |
| Beta blocker | Prescribed medication | 15,693 (22.9) | X | X | X | X |  |
| Bile acid sequestrant | Prescribed medication | 106 (0.1) |  |  |  |  |  |
| Calcium channel blocker | Prescribed medication | 16,493 (24.0) |  |  | X |  |  |
| Centrally acting antihypertensive | Prescribed medication | 699 (1.0) |  |  | X |  |  |
| Corticosteroid | Prescribed medication | 6,715 (9.8) |  |  | X |  |  |
| Diuretic | Prescribed medication | 24,114 (35.1) |  | X | X | X | **X** |
| DPP-4 inhibitors (Gliptins) | Prescribed medication | 319 (0.5) |  |  | X |  |  |
| Fibrates | Prescribed medication | 210 (0.3) |  |  | X |  |  |
| Glinide | Prescribed medication | 51 (0.1) |  |  |  |  |  |
| Glucagon-like peptide-1 (GLP-1) | Prescribed medication | 70 (0.1) |  |  | X |  |  |
| Hormone replacement therapy | Prescribed medication | 1,024 (1.5) |  |  |  |  |  |
| Immunosuppressant | Prescribed medication | 6,587 (9.6) |  |  | X |  |  |
| Inotrope | Prescribed medication | 3,347 (4.9) | X | X | X | X | **X** |
| Loop diuretic | Prescribed medication | 9,518 (13.9) |  | X | X | X | **X** |
| Metformin | Prescribed medication | 4,524 (6.6) |  | X | X |  |  |
| Nicotinic acid | Prescribed medication | 11 (0.0) |  |  |  |  |  |
| Nitrates | Prescribed medication | 1,571 (2.3) |  |  | X |  |  |
| Non-steroidal anti-inflammatory drugs | Prescribed medication | 17,579 (25.6) |  |  |  |  |  |
| Opioid | Prescribed medication | 26,910 (39.2) | X |  |  |  |  |
| Oral contraception | Prescribed medication | 231 (0.3) |  |  |  |  |  |
| Peripheral vasodilator | Prescribed medication | 180 (0.3) |  |  | X |  |  |
| Proton pump inhibitor | Prescribed medication | 18,515 (27.0) | X | X |  |  |  |
| RAAS inhibitor | Prescribed medication | 20,142 (29.3) |  |  | X |  |  |
| Sodium-glucose co-transporter-2 inhibitors | Prescribed medication | 21 (0.0) |  |  | X |  |  |
| Statin potency | Prescribed medication | Low: 2,440 (3.5)  Moderate: 12,511 (18.2)  High: 2,917 (4.2) |  | X | X | X | **X** |
| Sulfonylureas | Prescribed medication | 3,221 (4.7) |  |  | X |  |  |
| Thiazide diuretic | Prescribed medication | 16,505 (24.1) | X | X | X | X | **X** |
| Thiazolidinediones | Prescribed medication | 597 (0.9) |  |  | X |  |  |
| Vasodilator | Prescribed medication | 283 (0.4) |  |  | X |  |  |
| Warfarin | Prescribed medication | 3,696 (5.4) |  | X | X | X |  |

Haem.: haematological; RAAS: renin-angiotensin-aldosterone system

For cluster analysis, the following highly correlated redundant variables were excluded: atrial fibrillation, beta-blocker, chronic kidney disease, diabetes mellitus with no complications, HDL/LDL ratio, metformin, total cholesterol, type-2 diabetes mellitus, warfarin, and weight.
