# Supplementary material for: A population-based study exploring phenotypic clusters and clinical outcomes in stroke using unsupervised machine learning approach
Source: PLOS Digit Health. 2023 Sep 13;2(9):e0000334. doi: 10.1371/journal.pdig.0000334 (PMC10499205; doi:10.1371/journal.pdig.0000334)
Supplement: S2 Table — (DOCX) [file pdig.0000334.s009.docx]

**S2 Table. Observed versus imputed values after multiple imputation for all clinical variables with missing data**

| Variables | Median (Interquartile range) | |
| --- | --- | --- |
|  | Observed | Imputed |
| Alanine aminotransferase | 19.0 (15.0 – 27.0) | 23.2 (21.23 – 25.83) |
| Albumin level | 41.0 (38.0 – 43.0) | 40.6 (39.9 – 41.2) |
| Alkaline phosphatase | 82.0 (67.0 – 104.0) | 95.0 (89.1 – 102.5) |
| Bilirubin level | 10.0 (7.0 – 13.0) | 10.9 (10.1 – 11.8) |
| Body mass index | 26.3 (23.1 – 30.0) | 26.5 (25.6 – 27.4) |
| Diastolic blood pressure | 80 (71 – 85) | 80 (78 – 81) |
| Systolic blood pressure | 140 (130 – 150) | 141 (139 – 143) |
| Calcium level (adjusted) | 2.34 (2.27 – 2.41) | 2.34 (2.32 – 2.36) |
| Calcium level | 2.33 (2.26 – 2.41) | 2.34 (2.32 – 2.36) |
| Creatinine level | 87.0 (74.0 – 104.0) | 92.2 (88.6 – 97.0) |
| C-reactive protein | 5.0 (3.0 – 11.0) | 10.7 (7.3 – 15.3) |
| Eosinophil level | 0.2 (0.2 – 0.3) | 0.3 (0.2 – 0.4) |
| Erythrocyte sedimentation rate | 14.0 (7.0 – 27.0) | 18.5 (14.3 – 22.7) |
| Gamma glutamyl transpeptidase | 29.0 (19.0 – 51.0) | 44.9 (36 – 58.5) |
| Glomerular filtration rate | 66.0 (56.0 – 81.0) | 67.2 (64.0 – 70.5) |
| Haemoglobin level | 13.5 (12.4 – 14.6) | 13.5 (13.2 – 13.9) |
| Glycated haemoglobin (hba1c) level | 47.5 (40.0 – 59.6) | 50.1 (47.4 – 53.1) |
| HDL/LDL ratio | 3.5 (2.0 – 4.4) | 3.7 (3.4 – 4.0) |
| Height | 1.65 (1.58 – 1.73) | 1.67 (1.64 – 1.69) |
| High-density lipoprotein (HDL) cholesterol | 1.4 (1.1 – 1.7) | 1.5 (1.4 – 1.6) |
| Low-density lipoprotein (LDL) cholesterol | 2.9 (2.2 – 3.6) | 3.0 (2.8 – 3.2) |
| Lymphocyte count | 1.8 (1.4 – 2.4) | 3.2 (2.6 – 3.9) |
| Neutrophil count | 4.3 (3.4 – 5.6) | 4.9 (4.6 – 5.6) |
| Platelet count | 248.0 (200.0 – 302.0) | 248.0 (234.4 – 261.7) |
| Potassium level | 4.4 (4.1 – 4.7) | 4.4 (4.3 – 4.5) |
| Pulse | 76 (68 – 84) | 76 (74 – 79) |
| Sodium level | 140 (137 – 142) | 139 (138 – 140) |
| Thyroid stimulating hormone level | 1.8 (1.2 – 2.7) | 2.1 (2.0 – 2.3) |
| Total cholesterol level | 5.0 (4.2 – 5.8) | 5.1 (4.9 – 5.3) |
| Triglyceride level | 1.3 (1.0 – 1.8) | 1.5 (1.3 – 1.6) |
| Urea | 6.0 (4.8 – 7.6) | 6.4 (5.9 – 6.9) |
| Weight | 73.0 (61.6 – 85.0) | 74.2 (70.8 – 77.6) |
