# Supplementary material for: A population-based study exploring phenotypic clusters and clinical outcomes in stroke using unsupervised machine learning approach
Source: PLOS Digit Health. 2023 Sep 13;2(9):e0000334. doi: 10.1371/journal.pdig.0000334 (PMC10499205; doi:10.1371/journal.pdig.0000334)
Supplement: Attachment — Submitted filename: Response to Reviewers.docx [file pdig.0000334.s010.docx]

***Journal:***	*PLOS Digital Health*

***Manuscript #:***	*PDIG-D-23-00084R1*

***Title:***	*A population-based study exploring phenotypic clusters and clinical outcomes in stroke using unsupervised machine learning approach*

***Authors:***	*Ralph K. Akyea, George Ntaios, Evangelos Kontopantelis, Georgios Georgiopoulos, Daniele Soria, Folkert W. Asselbergs, Joe Kai, Stephen F. Weng, Nadeem Qureshi*

We greatly appreciate the overall assessment and feedback from the Reviewers. Our response to each of the reviewers’ comments and recommendations is detailed below.

**Journal Requirements**

Comment:

*“Please review your reference list to ensure that it is complete and correct. If you have cited papers that have been retracted, please include the rationale for doing so in the manuscript text or remove these references and replace them with relevant current references. Any changes to the reference list should be mentioned in the rebuttal letter that accompanies your revised manuscript. If you need to cite a retracted article, indicate the article's retracted status in the References list and also include a citation and full reference for the retraction notice.”*

Response:

The reference list has been reviewed as recommended. The references are correct and complete. No retracted paper has been cited in the manuscript.

**Response to Comments from Referee 1**

Comment:

*The study is well designed, and the manuscript is well written. The dataset and pre-processing steps are introduced carefully. Method and interpretable method is very beneficial for the research domain.*

Response:

Thank you for your review and for highlighting how the method and interpretable method are very beneficial for the research domain.

**Response to Comments from Referee 2**

Comment:

*This study offers a data-driven approach to stratify individuals with incident stroke into phenotypic clusters and identify the risk of major adverse outcomes. This is a very interesting approach that can help redefine patients risks.*

Response:

Thank you for your review and the positive feedback.

Comment:

*However, I have a few remarks:*

*Why did you choose a composite outcome for your primary outcome? Is this based on recommendations by clinicians? If so, you should explicit this choice in the methodology, as composite criteria are never ideal for this type of studies. However, if this choice is based on real-life experience (and thus, justified), you should explain it in the manuscript.*

Response:

Thank you for your feedback regarding our choice of a composite outcome for the primary outcome. The decision to use a composite outcome was based on clinical considerations and aligned with previous cluster analysis studies, such as the work of Verdonschot J. *et al*. published in the European Heart Journal (<https://doi.org/10.1093/eurheartj/ehaa841>).

Comment:

*While multiple imputation is the correct approach for missing data, I would like to see more info about this data from a clinician point of view: is this data missing because it’s not relevant for these type of patients (and maybe this variable should not be included in the model) or are the patients with missing data different from other patients? Since you are trying to identify patients with phenotypic similarities, the reason why this data is missing could be relevant.*

Response:

Electronic health records offer valuable data for clinical research, but it's important to acknowledge that missing data is a common issue in routinely collected health data. The information available in these records depends on the healthcare provider's input during the patient's routine care. Any data that hasn't been coded in the patient record will not be accessible for clinical research in the CPRD. Moreover, certain health information like smoking status, BMI, or ethnicity data may only be recorded when it's relevant to the patient's health condition, potentially introducing bias in data completeness patterns. To address this, multiple imputation of missing values is a well-established and recommended approach, taking into account demographic characteristics, disease status, and disease outcome (<https://doi.org/10.1186/S12874-016-0281-5>).

Information on missing data and the use of multiple imputation has now been added to the discussion section (see page 20):

*“The presence of missing data is a common occurrence in clinical research using electronic health records collected as part of routine care. For example, laboratory tests are typically requested only when considered necessary for a patient's health condition. Similarly, information on BMI or smoking status may not be consistently recorded, leading to potential bias in patterns of data completeness. To address this issue, multiple imputation by chained equations, as outlined in the methods section, was used to handle missing data in our study, which is the preferred option under any missingness mechanism. [19, 20]”*

References:

[19]	P. Royston, Multiple imputation of missing values: Update of ice, Stata J. 5 (2005) 527–536.

[20]	E. Kontopantelis, I.R. White, M. Sperrin, I. Buchan, Outcome-sensitive multiple imputation: a simulation study, BMC Med. Res. Methodol. 17 (2017) 1–13. <https://doi.org/10.1186/S12874-016-0281-5>.

Comment:

*Why did you use chi-squared tests for categorical data? Why not Fisher tests? You used a non-parametric test for continuous data and a parametric test for categorical data. I think you should either use non-parametric tests for both types of data or justify your methodological choice.*

Response:

Thank you for your feedback on the choice of statistical tests. We appreciate your suggestion to consider Fisher tests for categorical data. While Fisher tests are non-parametric and suitable for examining associations or testing independence between variables in categorical data, our study's sample size is sufficiently large and the assumptions for chi-squared tests are met. The Fisher test is generally preferred or recommended when sample sizes are small or when the assumptions of the chi-squared test are not met.

We chose the chi-squared test because it is widely accepted and provides reliable results in our research context. We understand that using non-parametric tests for continuous data and parametric tests for categorical data may appear inconsistent. However, this methodological choice was carefully made based on the nature of the variables and the research question. The non-parametric test was appropriate for our continuous data as it did not meet the assumptions of a parametric test.

Comment:

*It’s interesting to see that ethnicity does not seem to influence the outcome, even though previous studies have shown links between characteristics such as BMI or hypertension and ethnicity (higher hypertension amongst black people, cardiovascular risk with lower BMI in people of East Asian descent...) However, the number of non-white patients is low in the database, which could explain this result. It would be great to compare these results with databases with a majority of black patients or a majority of Asian patients to confirm that ethnicity does not have a big influence on the risk of subsequent major adverse outcomes. Unfortunately, I’m not sure this is feasible at this moment.*

Response:

Thank you for bringing this up. The data-driven approaches, Boruta and Least Absolute Shrinkage and Selection Operator (LASSO) regression have identified ethnicity as an important feature for cluster analysis. However, since the data is based on real-world electronic health records from UK primary care, the low number of non-White patients is due to the dataset's representation of the UK general population. Unfortunately, comparing the results within different ethnic groups is not feasible due to the limited numbers. Nevertheless, this approach can be applied to other datasets from different settings. It presents an opportunity to replicate the study in an Asian cohort, for example. The conclusion section highlights the need for further exploration/work in different patient cohorts and populations (page 21).
